# Redrawing the Iceland−Scotland Overflow Water pathways in the North Atlantic

**DOI:** 10.1038/s41467-020-15513-4

**Published:** 2020-04-20

**Authors:** Sijia Zou, Amy Bower, Heather Furey, M. Susan Lozier, Xiaobiao Xu

**Affiliations:** 1 Woods Hole Oceanographic Institution, Woods Hole, MA USA; 2 Georgia Institute of Technology, Atlanta, GA USA; 3 Center for Ocean-Atmosphere Prediction Studies, Florida State University, Tallahassee, FL USA

**Keywords:** Physical oceanography, Physical oceanography

## Abstract

Iceland-Scotland Overflow Water (ISOW) is a primary deep water mass exported from the Norwegian Sea into the North Atlantic as part of the global Meridional Overturning Circulation. ISOW has historically been depicted as flowing counter-clockwise in a deep boundary current around the subpolar North Atlantic, but this single-boundary-following pathway is being challenged by new Lagrangian observations and model simulations. We show here that ISOW leaves the boundary and spreads into the interior towards the central Labrador and Irminger basins after flowing through the Charlie-Gibbs Fracture Zone. We also describe a newly observed southward pathway of ISOW along the western flank of the Mid-Atlantic Ridge. The partitioning of these pathways is shown to be influenced by deep-reaching eddies and meanders of the North Atlantic Current. Our results, in tandem with previous studies, call for a revision in the historical depiction of ISOW pathways throughout the North Atlantic.

## Abstract (web summary)

Global ocean circulation overturns and starts anew in the North Atlantic, propagating climate signals to the rest of the oceans. Using drifter data, Zou and colleagues re-map the spreading pattern for one of the deep water masses and show the impact of mesoscale processes on that pattern.

## Introduction

Iceland−Scotland Overflow Water (ISOW), together with Denmark Strait Overflow Water and Labrador Sea Water, are transported away from their formation sites in the Nordic Seas and subpolar North Atlantic by the lower limb of the Atlantic Meridional Overturning Circulation (AMOC). The spreading pathways of these deep waters are important not only for understanding the geographic structure of the AMOC but also for transporting high-latitude climate signals, such as anthropogenic carbon, to the rest of the ocean. Compared to the other two water masses, the spreading pathways of ISOW are less well-known.

**Fig. 1:**
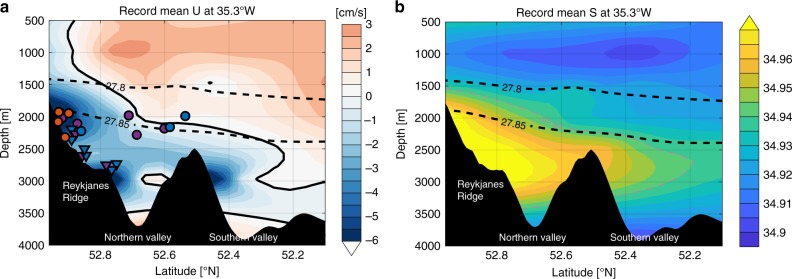
Vertical sections across the Charlie−Gibbs Fracture Zone (~35.3°W). **a** Zonal mean velocity from mooring observations. Superimposed are the 21 RAFOS floats’ positions while crossing through/nearby the section. Triangles show release locations for nine floats in the northern valley and circles show locations of the remaining 12 floats while passing through/nearby the section. Marker color indicates the pathway each float took: blue for west-northwestward pathway; purple for southward pathway; and orange for eastward pathway. **b** Mean salinity from the same source. On both plots, dashed black contours show isopycnals.

**Fig. 2:**
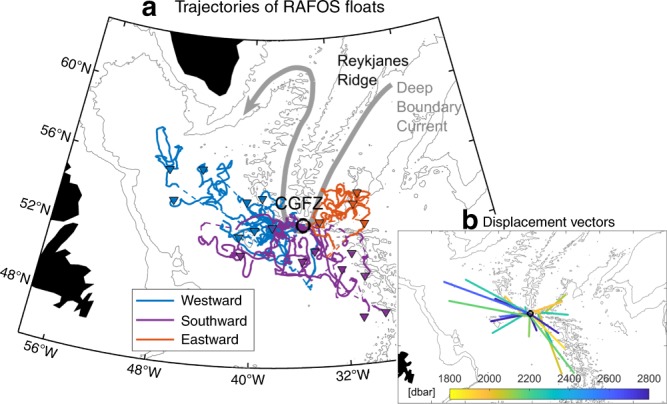
ISOW pathways from the Charlie−Gibbs Fracture Zone. **a** Trajectories of 21 RAFOS floats whose initial (final) locations are shown as a black open circle (colored triangles). The three major pathways (see Methods) are illustrated with different colors: blue for west-northwestward pathway; purple for southward pathway; and orange for eastward pathway. Dashed trajectory segments indicate missing tracks. **b** Displacement vectors from the Charlie−Gibbs Fracture Zone to floats’ surface positions, color coded by mean along-track pressure. On both plots, the 2000- and 3000-m isobaths are contoured in gray.

ISOW spills into the North Atlantic from the Norwegian Sea between Iceland and Scotland. Entrainment of saltier Subpolar Mode Water along its descent into the Iceland Basin differentiates ISOW from the fresher Denmark Strait Overflow Water and Labrador Sea Water throughout the subpolar North Atlantic^1–3^. ISOW has historically been depicted in schematic circulation diagrams as flowing counter-clockwise in the deep boundary current around the rim of the subpolar North Atlantic. This path begins with a southwestward leg along the eastern flank of the Reykjanes Ridge as far south as the Charlie−Gibbs Fracture Zone (CGFZ), which is the major gateway for ISOW to flow from the eastern to western North Atlantic^4,5^ (Fig. 1). West of the CGFZ, most circulation diagrams show that ISOW turns sharply northward to follow the bathymetry of the western flank of the Reykjanes Ridge, and continues counter-clockwise around the Irminger Sea^6,7^ (gray curve in Fig. 2a).

In the past three decades, the spreading pathways of ISOW have been found to be more complex than the single deep boundary current path. For example, Lagrangian observations, hydrographic sections and model simulations show that the shallower component of ISOW east of the Reykjanes Ridge flows westward through deep gaps in the ridge before reaching the latitude of the CGFZ^8–11^. In addition, Lagrangian floats, tracer and direct current observations show some ISOW by-passing the westward passage through CGFZ and instead continuing southward along the eastern flank of the Mid-Atlantic Ridge (MAR) into the Western European Basin^10,12–14^.

West of the CGFZ, a number of studies have suggested that not all ISOW turns northward along the western flank of the Reykjanes Ridge to flow counter-clockwise around the rim of the Irminger Sea. Hydrographic sections in the western subpolar North Atlantic have revealed a tongue of higher-salinity water extending west-northwestward from the CGFZ at the ISOW level, suggesting an alternative westward spreading of ISOW^15–18^. Daniault et al.^18^ further argued that ISOW emerging from the CGFZ first spreads westward and then turns northward into the central Irminger Sea. This pathway is illustrated with more details in a modeling study^9^, which pointed out a deep ($\sigma _\theta$ > 27.85 kg m^−3^) mean west-northwestward current that extends from the CGFZ nearly to the southern tip of Greenland. The study further shows that in the model, most of the ISOW passing through the CGFZ follows this path, with a much lower fraction turning northward into the Irminger Sea. More recently, Racapé et al.^19^ reported that one of the five Deep-Argo floats initialized in the CGFZ followed a direct westward route from the CGFZ to the Flemish Cap, where it was able to join the Deep Western Boundary Current.

Within the CGFZ, both shipboard LADCP measurements and moored observations show ISOW transport reversals, revealing a tight linkage between the westward ISOW transport and the position of the eastward-flowing North Atlantic Current (NAC)^5,7^. It remains unclear whether the NAC also impacts the ISOW spreading pathways downstream (west) of the CGFZ.

In order to obtain a more comprehensive description of the ISOW spreading pathways from the CGFZ and to disentangle the impact of the NAC on those pathways, here we present new Lagrangian observations and model results that support the emerging view of alternative ISOW pathways. In contrast to historical depictions, we show that the majority of ISOW travels west-northwestward along interior pathways into the central Labrador and Irminger Seas, similar to previous hydrographic evidence and modeling studies. In addition, we describe a surprising southward branch that flows along the western flank of the MAR. This sizeable southward branch is shown to potentially serve as a fast track for some ISOW to export to the subtropical latitudes. Finally, we reveal the influence of the deep-reaching eddies and meanders that are associated with the NAC on the ISOW spreading pathways.

## Results

### Observed ISOW pathways from the CGFZ

Twenty-one neutrally buoyant, acoustically tracked isobaric RAFOS (Range and Fixing of Sound) floats^20^, deployed at ISOW levels (1800−2800m) as part of the Overturning in the Subpolar North Atlantic Program^21^, are analyzed here. Nine of these floats were released directly in the northern valley of the CGFZ on June 28, 2014 (triangles in Fig. 1). Twelve were deployed along the Reykjanes Ridge’s eastern flank and drifted through or nearby the CGFZ between 2014 and 2018 (circles in Fig. 1). Mooring observations of the mean velocity and salinity fields across the CGFZ are also shown in Fig. 1, which are adapted from Bower and Furey^5^ with permissions. The floats’ lifetime varies in length from 78 to 730 days, with a mean of 498 days. Detailed information about the floats can be found in Supplementary Table 1. All of the floats passed by the northern valley of the CGFZ (i.e. north of 52.5°N) with in situ temperatures between 2.9 and 3.3 °C. These temperatures indicate that the floats were in the ISOW layer, which, according to mooring observations^5^, is characterized by densities greater than 27.80 kg m^−3^, salinities greater than 34.94, and in situ temperatures from 2.4 to 3.4 °C. It is worth noting that the launch time of the RAFOS floats is not within the temporal span of the mooring data (2010−2012); thus, the properties across the section at launch likely differ. However, the general identification of ISOW is robust.

The floats’ trajectories and net displacements are displayed in Fig. 2. Of the 21 floats, eight exhibited quasi-unidirectional large-scale motion towards the west-northwest, and three of those made it as far as the central Labrador Sea. Surprisingly, another set of eight floats flowed southward along the western flank of the MAR. These floats surfaced south of the CGFZ, with two reaching as far south as 47°N. This southward spreading is independent of float depth or launch position (purple markers in Fig. 1a). Four floats of the total recirculated eastward towards the Iceland Basin. These floats were launched at shallower depths (~2000 m) than most of the other floats (orange markers in Fig. 1a). We surmise that their relatively shallow depths made them more likely to be carried eastward by the deep-reaching NAC, which is confirmed by simulated floats, as discussed below. Among the 21 floats, only one had the potential to follow the deep boundary current by turning northward along the western flank of the Reykjanes Ridge. However, this float stopped its mission at 53.3°N at which point it had become tangled in the higher peaks of the ridge (Supplementary Fig. 1).

Although few in number, these float observations contradict the historical view that ISOW is systematically transported northward along the western flank of the Reykjanes Ridge after entering the western subpolar gyre through the CGFZ (i.e. along the deep boundary current), as depicted in many circulation schematics.

**Fig. 3:**
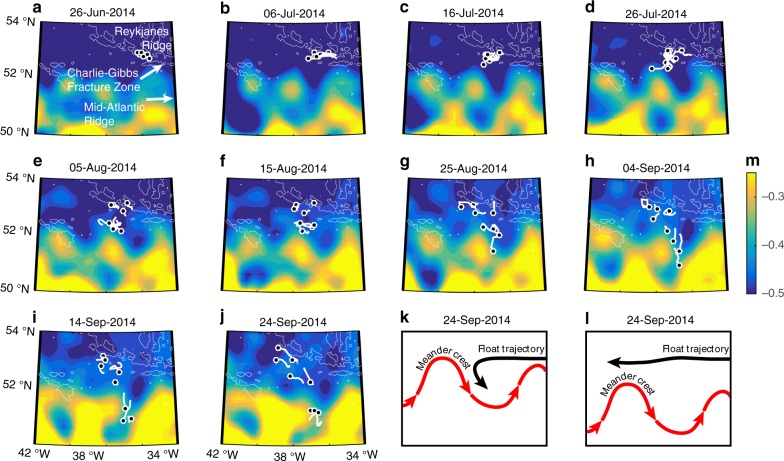
Interactions between ISOW paths and eddy/meandering activities. **a**–**j** Time sequence of absolute dynamic topography maps at 10-day intervals from AVISO starting in June 2014, with 10-day-long RAFOS float trajectory segments superimposed as white short lines. The final location for each float segment is indicated as a black dot. The 2000- and 3000-m isobaths are shown in white. **k** Schematic of the interaction between a float trajectory and a northward-shifted meander crest. **l** Schematic of the interaction between a float trajectory and a southward-shifted meander crest.

The westward spreading of ISOW from the CGFZ is consistent with previous hydrographic measurements^15,16^. Modeling study^9^ suggests that this westward pathway may be due to a deep mean west-northwestward current that extends from the CGFZ to the southern tip of Greenland (also see Supplementary Fig. 5). However, the southward pathway along the western flank of the MAR has, to our knowledge, never been observed or reported. What determines this southward spreading pathway of ISOW? To answer this question, we plot 10-day track segments of the eight floats (excluding the one float that turned slightly northward) that were deployed in the CGFZ on June 28, 2014 (triangles in Fig. 1a) and superimpose them with a time sequence of absolute dynamic topography (ADT) maps from AVISO (Fig. 3) that show the strength and location of major surface current branches. The floats roughly split into two groups with different behavior: one set followed the 3000 m isobaths and drifted toward the west-northwest, while the other encountered meanders and/or eddies associated with the NAC (evident from higher ADT gradients). These latter floats were first trapped at the crests of the eddies/meanders (i.e. the northernmost positions) and were then diverted southward by the anticyclonic flow, roughly parallel to the ADT contours (Fig. 3g-j). A more detailed example of the ADT’s impact on the southward float dispersion can be found in Supplementary Fig. 2.

**Fig. 4:**
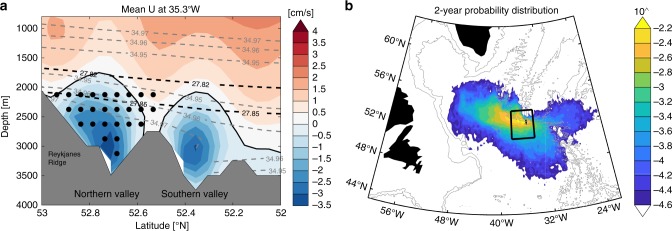
Simulated ISOW paths from the Charlie−Gibbs Fracture Zone. **a** Modeled zonal mean velocity at 35.3°W from 1990 to 2004. The zero-velocity is contoured in black. Mean isopycnals (isohalines) are contoured in dashed black (gray). Black circles represent the synthetic float launch locations. **b** Probability distribution of 2-year simulated particle trajectories after their initialization (see Methods). The probabilities shown here are on a log scale. Black box ([38°W, 34°W], [51°N, 54°N]) denotes the area where eddy kinetic energy is calculated. The 2000- and 3000-m isobaths are contoured in gray.

The sequence shown in Fig. 3 suggests that at least to some extent, eddies and meanders associated with the NAC extend to the depth of ISOW and influence the ISOW pathways from the CGFZ. For example, when eddies and meanders are active west/southwest of the CGFZ due to a northward shift of the NAC, ISOW may be pulled off its westward path and travel southward, resulting in a stronger southward spreading (Fig. 3k). When the NAC’s eddies and meanders are less active at the CGFZ due to a southward shift of the NAC, we surmise that ISOW may be more likely to continue its westward spreading, with less southward dispersion (Fig. 3l). The interaction between ISOW transport and the NAC was shown to be the case within the CGFZ based on the 2-year moored array observations^5^ and in a follow-up modeling study^9^. To test whether the NAC also interacts with ISOW spreading pathways west of the CGFZ, we turn to output from a high-resolution numerical model, FLAME (see Methods). A simulation of ISOW spreading pathways with another eddy-resolving model (HYCOM) reveals differences in the details compared to the FLAME results, but overall the simulations from the two models show consistent spreading patterns (Supplementary Fig. 10). We have chosen to show the results from FLAME because it exhibits transport structure in the northern valley of the CGFZ (Fig. 4a) more compatible with observations (Fig. 1a). In addition, it shows more favorable statistical quantification of the simulated ISOW pathways when compared to the RAFOS observations. Note that these results may be related. One caveat about the use of FLAME is that its temporal span does not overlap with that for the RAFOS floats. As such, there may be some differences in the NAC position and/or strength between the model and observations, yet these differences do not impact our overall description of ISOW pathways, as illustrated below.

### Simulated ISOW pathways from the CGFZ

To test whether FLAME adequately simulates the ISOW spreading pathways, we released 3593 synthetic floats monthly from 1990 to 2004 at ISOW levels in the northern valley of the CGFZ (at 35.3°W; Fig. 4a). The floats were then integrated forward in time using three-dimensional velocity fields for 2 years (which is the length of the longest observed float trajectory). The probability distribution of the simulated trajectories is shown in Fig. 4b. Consistent with the observations, three dominant spreading branches are identified: a major branch travels west-northwestward to the central Labrador Sea and Irminger Sea; one branch moves southward along the western flank of the MAR; and another branch recirculates and spreads eastward to the eastern North Atlantic. In addition to these branches, a number of floats recirculate in the vicinity of the CGFZ, apparently trapped by eddies. An example of these four major branches can be found in Supplementary Fig. 3.

The simulated ISOW branches are further quantified based on the floats’ spreading pathways and surface locations (see Methods). Of all 3593 simulated floats, 59 ± 9% follow the west-northwestward interior pathway. These floats are preferentially initialized in the deepest ISOW layer (below 2600 m) in the CGFZ (Supplementary Fig. 4a). The dominant west-northwestward pathway follows a deep mean northwestward current that connects the CGFZ to the central Irminger and Labrador Seas (Supplementary Fig. 5). 19 ± 8% of the floats take the southward pathway west of the MAR, with no discernible preference of their initial positions in the CGFZ (Supplementary Fig. 4b). The floats that travel eastward, accounting for 6 ± 5%, primarily originate from the shallowest ISOW layer in the CGFZ (Supplementary Fig. 4c), consistent with the observations. 9 ± 6% of the floats circulate locally near the CGFZ and their initial locations preferentially concentrate to the northern part (i.e. close to the ridge) of the northern valley (Supplementary Fig. 4d). Only 5 ± 4% of the floats follow the deep boundary current (between the 2000 and 3000 m isobaths) continuously from the CGFZ to the Irminger Sea along the western flank of the Reykjanes Ridge (Supplementary Fig. 6). These boundary-following floats are concentrated in the northern part of the northern valley at initialization. Compared to the observed float pathway percentages (8/21 or 38% for both the west-northwestward and the southward pathways), the model appears to overestimate the west-northwestward branch and underestimate the southward branch. Such a discrepancy is partially attributed to the low number of observed floats that precludes a rigorous statistical comparison. Another attribution is that the observed floats were traveling during a time (2014−2016) with stronger eddy/meandering activities west of the CGFZ (Supplementary Fig. 7). As discussed below, stronger eddy/meandering activities may lead to suppressed westward spreading and enhanced southward spreading of ISOW.

### Impact of the NAC on the ISOW pathways

**Fig. 5:**
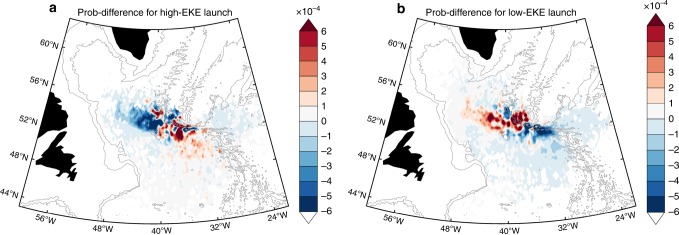
Impact of eddies/meanders on the simulated ISOW pathways. **a** The difference in the 2-year probability distribution between 509 floats that traveled during high eddy kinetic energy periods and all floats. **b** The difference in the 2-year probability distribution between 752 floats that traveled during low eddy kinetic energy periods and all floats. In both plots, the 2000- and 3000-m isobaths are contoured in gray.

As stated above, we suspect that the eddies and meanders of the NAC may influence the ISOW spreading pathways. To test this supposition, we calculate the eddy kinetic energy (EKE) west of the CGFZ (black box in Fig. 4b) from the model velocity fields to approximate the activity of the eddy/meandering field in the region. The EKE time series near the surface (10 m) and at the ISOW level (2500 m) are significantly correlated (*r* = 0.60 for monthly time series and *r* = 0.84 for annually averaged time series; Supplementary Fig. 8). The good comparison between the two time series suggests that the NAC’s impact extends from the surface to the deep ocean and that surface EKE is a good proxy for deep EKE. Based on the surface EKE time series, we select simulated floats that traveled during high EKE periods and low EKE periods (see Methods) and compare the resultant spreading patterns with Fig. 4b. When EKE is high west of the CGFZ, fewer floats follow the west-northwestward branch, and more floats spread to the south (Fig. 5a). We re-calculate the pathway percentage with the high-EKE floats only and find that the mean percentage for the west-northwestward interior branch decreases to 48 ± 11% while the percentage for the southward branch increases to 31 ± 10%. The percentage differences for the eastward, local and deep boundary current branches are not significant (<3%). On the contrary, when EKE is low west of the CGFZ, more floats follow the westward branch (68 ± 10%) and fewer travel southward (12 ± 7%) (Fig. 5b).

These results support the idea that the NAC’s eddy/meandering activities can influence the spreading pattern of ISOW emerging from the CGFZ. Specifically, when the eddy/meandering field is active southwest of the CGFZ (e.g. when the NAC shifts further north), ISOW can be trapped at the eddy/meander crest, where the associated anticyclonic flow pulls ISOW off the westward pathway and diverts it southward. When the eddy/meander field is less active southwest of the CGFZ, ISOW is more likely to travel westward. Such an interaction between the NAC and ISOW pathways west of the CGFZ is similar to the interaction between the NAC and ISOW transport within the CGFZ^5,7^.

### Simulated ISOW pathways over 10 years

**Fig. 6:**
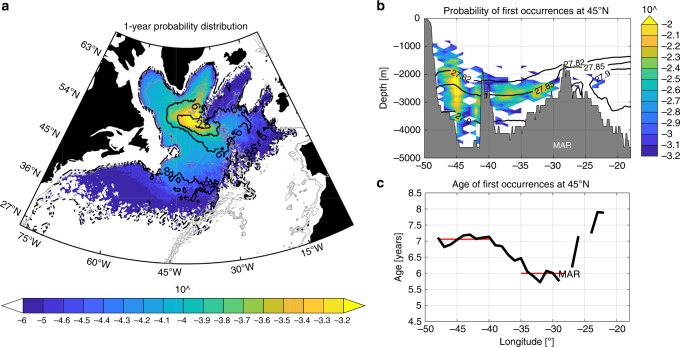
Simulated ISOW pathways over 10 years. **a** Probability distribution (on a log scale) of 10-year simulated particle trajectories after initialization in the Charlie−Gibbs Fracture Zone. Black contours represent the floats’ mean age (see Methods). **b** Probability distribution of first occurrences at 45°N. Mean isopycnals are contoured in black. **c** Age of the floats’ first occurrences at 45°N as a function of longitude (see Methods). The mean ages from the western boundary to 40°W (7.1 years) and along the western flank of the Mid-Atlantic Ridge (35°W−28°W; 5.9 years) are also plotted as red solid lines.

The spreading pattern for ISOW from the CGFZ is examined for a longer time period using simulated trajectories. Figure 6a shows the probability distribution of simulated trajectories integrated for 10 years after launch, with the floats’ mean age contoured. Within 10 years, float trajectories spread throughout the subpolar North Atlantic with some reaching as far south as ~30°N. Floats that are exported from the subpolar to the subtropical region are mostly concentrated west of the MAR, according to the probability distribution of floats’ first occurrences at 45°N, a latitude that approximates the boundary between the subpolar and the subtropical regions (Fig. 6b). Several export branches are identified at this latitude. One major export branch is along the Deep Western Boundary Current (i.e. west of ~44°W), and another weaker branch is between 44°W and 35°W, the latter denoting interior pathways. The mean age of floats for these branches is 7.1 ± 2.0 years west of 40°W and decreases eastward (Fig. 6c). These two export branches (i.e. the Deep Western Boundary Current and the interior pathway) are primarily taken by floats that follow the west-northwestward pathway from the CGFZ (Supplementary Fig. 9). A portion of these west-northwestward spreading floats turn northeastward south of Greenland and circulate back to the eastern Irminger Sea, where some of them join the cyclonic circulation that carries them around the basin and into the Labrador Sea (Supplementary Fig. 9a). Together with the floats that directly enter the Labrador Sea from the CGFZ, these floats are exported downstream to the subtropical region via both Deep Western Boundary Current and interior pathways (Supplementary Fig. 9c).

Another major export branch at 45°N is found along the western flank of the MAR (35°W−28°W). This branch contains the floats that move southward directly from the CGFZ (Supplementary Fig. 9d). The mean age of floats in this branch is 5.9 ± 1.9 years. The overall shorter time floats take from the CGFZ to 45°N along the western MAR suggests that this southward branch may be a faster track for ISOW to export to the subtropical region.

Collectively, these results suggest that the ISOW spreading pattern and export time scale depend on the partitioning between the west-northwestward branch and the southward branch west of the CGFZ. This partitioning, as discussed above, is in turn dependent on the eddy/meandering activities associated with the NAC.

## Discussion

With results from observed and simulated floats, we re-draw the major pathways of ISOW coming through the CGFZ: instead of turning northward along the western flank of the Reykjanes Ridge, most of the ISOW either follows a west-northwestward path or travels along a newly identified southward path along the western flank of the MAR. How can these new results be reconciled with the hydrographic signatures of ISOW found in the boundary current west of the Reykjanes Ridge^6^? The answer likely lies in the growing evidence that some ISOW leaks through gaps in the Reykjanes Ridge north of the CGFZ, including the Bight Fracture Zone at ~57°N^9–11,18^. This is a subtle but important distinction that was not clear before higher-resolution hydrography, Lagrangian observations and simulations were available.

This study suggests a new circulation scheme for ISOW in the North Atlantic—rather than moving along a simply connected boundary current, ISOW instead takes various interior routes that depend on the variability of the NAC. These emerging interior pathways for ISOW, together with those for other North Atlantic Deep Waters (e.g. the Labrador Sea Water^22^), reshape our view of how climate signals are exported from the high latitude North Atlantic to other ocean basins. Specifically, we now understand that the export is more diffusive than previously thought, a change with implications for how we measure inventories of heat, carbon and other tracers in the deep ocean.

The influence of NAC eddies and meanders on the deep ISOW spreading pathways is an unexpected result and indicates a connection between upper ocean mesoscale processes and the deep water transport variability. Interestingly, a recent modeling study has reported a similar connection in the South Atlantic: the zonal pathway of the North Atlantic Deep Water at 25°S is impacted by Agulhas rings through vorticity adjustment^23^. With a tight linkage between the deep water transport and the global Meridional Overturning Circulation, it is suggested that at least part of the overturning circulation variability on interannual time scales is associated with upper ocean mesoscale dynamics.

## Methods

### RAFOS float data

The 21 RAFOS floats presented in this study are a subset of a larger float data set (125) coming out of the Overturning in the Subpolar North Atlantic Program (OSNAP)^21^ float program: a basin-wide Lagrangian study of the spreading pathways of dense overflow waters that make up the lower limb of the AMOC.

The RAFOS floats examined here were all ballasted for the ISOW layer, that is, where $\sigma _\theta \ge$ 27.80 kg m^−3^ and high in salinity, specifically at depths between 1800 and 2800 m. The objective was to release the floats in the ISOW 100−200 m above the seafloor, where the strongest current speeds and highest salinities are typically found^5,24^. All OSNAP floats were tracked underwater using an array of 10 (13) moored sound sources during 2014−2016 (2016−2018). The floats recorded acoustic times of arrival, pressure and temperature once per day. The times of arrival are converted to distance and the float trajectories are reconstructed after the floats surface, in this case, 2 years after deployment. Supplementary Table 1 lists the technical information for each of the 21 floats analyzed here. Some floats exhibited missing tracks, which usually took place in winters due to rough conditions and/or topographic blocking of signals from the sound sources.

### Model FLAME and trajectory configuration

To extend the Lagrangian analysis in both the temporal and spatial domain, we simulate ISOW spreading pathways using an eddy-resolving (1/12°) ocean circulation model. The model is a member of the Family of Linked Atlantic Models Experiment (FLAME)^25,26^, and is forced with monthly anomalies of NCEP/NCAR reanalysis^27^ after a 10-year spin-up by the European Center for Medium-Range Weather Forecast climatological forcing. The model has a horizontal spatial domain from 18°S to 70°N on a Mercator grid and 45 vertical levels, ranging from 10 m near the surface to 250 m near the ocean bottom.

A number of previous studies have demonstrated FLAME’s ability to simulate the properties and spreading pathways of the deep waters in the North Atlantic^22,28–30^. Specifically, the model has been shown to produce volume transports and spreading pathways of ISOW in the eastern subpolar North Atlantic that are compatible with a suite of observations in the region, as well as with results from other modeling studies^10^. In the CGFZ, the mean transport in the ISOW layer is −0.9 Sv in FLAME, with an annual standard deviation of 0.4 Sv. This simulated transport is smaller than that in observations (−1.7 Sv in Bower and Furey^5^; −2.4 Sv in Saunders^4^), indicating a weaker long-term mean or an underestimated ISOW branch through the CGFZ in FLAME. We note however that the ISOW transport through the CGFZ is highly variable on mesoscale time scales in both moored observations and models, making it difficult to define the long-term mean accurately (especially with limited observational time series).

The model output used in this study is temperature, salinity, and three-dimensional velocity field at a 3-day temporal resolution from 1990 to 2004. ISOW across the CGFZ (35.3°W, 52−53°N) is defined with both density and salinity. The modeled density threshold (27.82 kg m^−3^) is slightly larger than the threshold from observations^5^ (27.80 kg m^−3^) because the model’s water masses are generally saltier than observed. The salinity threshold varies by year and is chosen by a careful examination of the property and velocity fields. Before and for 1995, the salinity threshold is 34.95; after and for 1996, the threshold is 34.96. Simulated floats are initiated only when the water is denser and saltier than their respective thresholds. After initiation, float trajectories are calculated using the three-dimensional velocity fields, as detailed in Gary et al.^29^. To extend the lifetime of floats launched in the last few years of the model duration, the model velocity fields are recycled with a single discontinuity between December 31, 2004 and January 1, 1990.

### Calculations of probability and age distributions

To calculate the probability distribution for simulated float trajectories, we first divide the North Atlantic into 0.25° × 0.25° grids and count the number of times floats pass through each grid (including repetitions). Then we divide the number of passes in each grid by the total float passes over all grids.

The mean age for floats in each grid box is computed by averaging the time elapsed since launch for each float, including repeated visits. At specific latitude (e.g. 45°N), the mean age at each longitude is calculated by averaging the time of their arrivals at this latitude within the longitude bin (1°).

### The selection of floats during high/low EKE periods

For each simulated float, we first identify its released month and then we compute the 10-m-depth EKE west of the CGFZ (black box in Fig. 4b) in the following 12 months. For example, if a float is released in May 1990, the EKE field will be calculated from May 1990 to April 1991. If the 12-month mean EKE is greater than the 15-year averaged EKE plus 1 standard deviation (i.e. 0.022 m^2^ s^−2^ at 10 m; also see Supplementary Fig. 8), this float is considered as traveling during a high EKE period. If the 12-month EKE is smaller than 15-year averaged EKE minus 1 standard deviation (i.e. 0.015 m^2^ s^−2^ at 10 m), the float is considered as traveling during a low EKE period. For all 3593 floats, 509 floats travel during high EKE periods and 752 floats travel during low EKE periods.

### Quantification of simulated ISOW spreading pathways

To quantify the simulated ISOW branches, we categorize the floats into five groups based on their spreading pathways and surface locations. The first group follows the west-northwestward pathway and ends up in the western subpolar gyre (west of the Reykjanes Ridge and north of 52°N). The second group travels southward with the floats’ final locations south of 52°N and west of the MAR. The third group is carried eastward into the eastern subpolar gyre and the fourth group circulates locally near the CGFZ (within [38°W, 32°W], [52°N, 54°N]). An example of these four groups is shown in Supplementary Fig. 3.

The last group includes the floats that follow the historical boundary current schematic. That is to say, the group includes floats that follow the deep boundary current continuously from the CGFZ northward along the western flank of the Reykjanes Ridge, which is characterized as the path between 2000 and 3000 m isobaths. The trajectories of this group are shown in Supplementary Fig. 6.

Of all 3593 simulated floats, a subsample of 21 floats is randomly selected and their trajectories are categorized based on the above criteria. [Note that the subsample size is chosen to match the number of the RAFOS floats.] This process is repeated for 10,000 times to derive the mean and the uncertainty of each group’s percentage.

## Supplementary information

Supplementary Information

## Data Availability

RAFOS float data can be accessed at Woods Hole Open Access Server (10.26025/1912/24388). FLAME model outputs are obtained from C. Böning and A. Biastoch at GEOMAR—Helmholtz-Zentrum für Ozeanforschung Kiel. HYCOM model outputs used in the supporting materials can be accessed on request to X.X. The altimeter products used here were produced by Ssalto/Duacs and distributed by AVISO, with support from CNES (http://www.aviso.altimettry.fr/duacs/). The CGFZ mooring data presented in this paper are available from NCEI (http://accession.nodc.noaa.gov/0164585).

## Code availability

The code used to compute the simulated trajectories and the quantification of the float pathways can be accessed upon request to S.Z.
