# Supplementary material for: Redrawing the Iceland−Scotland Overflow Water pathways in the North Atlantic
Source: Nat Commun. 2020 Apr 20;11:1890. doi: 10.1038/s41467-020-15513-4 (PMC7170894; doi:10.1038/s41467-020-15513-4)
Supplement: Supplementary file 1 — Supplementary Information [file 41467_2020_15513_MOESM1_ESM.pdf]

Supplementary Information for **Redrawing the Iceland-Scotland Overflow Water Pathways in the North Atlantic** by Zou et al.

Supplementary Table 1. Basic information for the 21 RAFOS floats. The launch and surface information for each float, denoted by a serial number, is reported. Duration days are defined as the days from launch date, if launched in Charlie-Gibbs Fracture Zone (CGFZ), or first date in CGFZ, if launched in the Iceland Basin, to their surface date. The surface location relative to the CGFZ is also indicated: W-west of the CGFZ; E-east of the CGFZ; N-north of the CGFZ; and S-south of the CGFZ.

float serial number	Launch or at CGFZ					Surface		Duration (Days)	Surface location W/E/N/S of CGFZ
	date (yyyymmdd)	Lat (°N)	Lon (°W)	pressure (dbar)	*in situ* temp (°C)	Lat (°N)	Lon (°W)		
Deployed in CGFZ (9)									
1319	20140628	52.878	35.727	2194.3	3.138	53.352	36.042	180	N
1321	20140628	52.866	35.718	2270.5	3.120	51.126	40.914	730	S
1322	20140628	52.909	35.640	2263.2	3.140	55.788	44.729	730	W
1333	20140628	52.863	35.458	2605.3	3.101	51.029	31.522	730	S
1334	20140628	52.873	35.484	2601.1	3.103	52.339	39.595	730	W
1338	20140628	52.867	35.482	2600.9	3.103	55.552	48.134	730	W
1341	20140628	52.795	35.350	2928.9	3.058	52.174	41.191	730	W
1342	20140628	52.762	35.305	2922.8	3.063	54.088	40.522	152	W
1344	20140628	52.793	35.344	2918.6	3.067	51.300	34.183	730	S
Deployed in Iceland Basin and reached the CGFZ (12)									
1316	20150905	52.872	35.368	2117.1	3.115	50.851	35.472	321	S
1326	20150614	52.687	34.101	2281.4	3.089	50.270	32.071	403	S
1329	20160505	52.871	35.459	2223.1	3.179	52.718	38.114	78	W
1357	20160412	52.536	36.169	1988.9	3.246	54.300	39.068	441	W
1365	20160411	52.712	35.151	1980.0	3.290	49.792	30.199	442	S
1378	20151208	52.601	35.318	2183.4	3.299	47.672	28.838	567	S
1379	20160412	52.584	35.606	2160.8	3.185	53.895	47.382	440	W
1398	20170213	52.932	32.998	2079.2	3.157	54.409	30.080	515	E
1402	20170417	52.959	34.080	2022.3	2.905	47.578	30.620	453	S

1406	20170325	52.914	34.010	2319.6	3.064	52.694	29.724	473	E
1438	20171028	52.899	35.109	1947.3	3.137	53.821	30.578	441	E
1439	20171030	52.918	34.537	1926.3	3.099	52.928	33.815	439	E

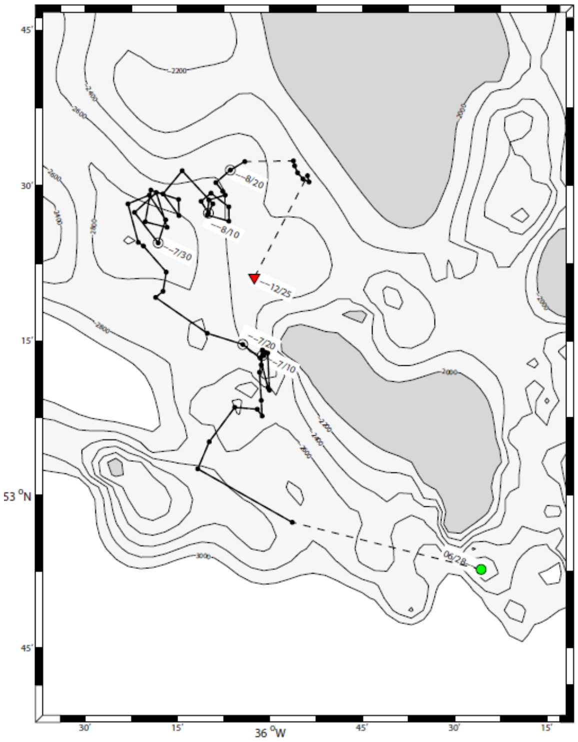

Supplementary Figure 1. Trajectory of one RAFOS float that turned northward. Zoomed in map showing the trajectory of float 1319, the only float that turned northward after being released in the Charlie-Gibbs Fracture Zone in June 2014. Dots along the trajectory are shown daily. Isobaths are drawn every 200 meters. Black dashed line segments indicate missing track. Date labels (mm/dd) mark positions at launch and surface, as well as on the last day of each month. Within about 2.5 months after release, the float started to get tangled up in the higher peaks of the Reykjanes Ridge, causing gaps in the tracking that got worse with time. It was a programmed short-mission (test) float, and surfaced 180 days after deployment, only ~67 km away from its deployment site—it is assumed from the severe loss in tracking and the float’s pressure record (not shown) that it grounded along the ridge crest for a significant portion of its 180-day mission. The divergence of 1319 from its partner floats illustrates the small spatial scales of the flow here, most likely introduced by the rugged volcanic-origin bathymetry.

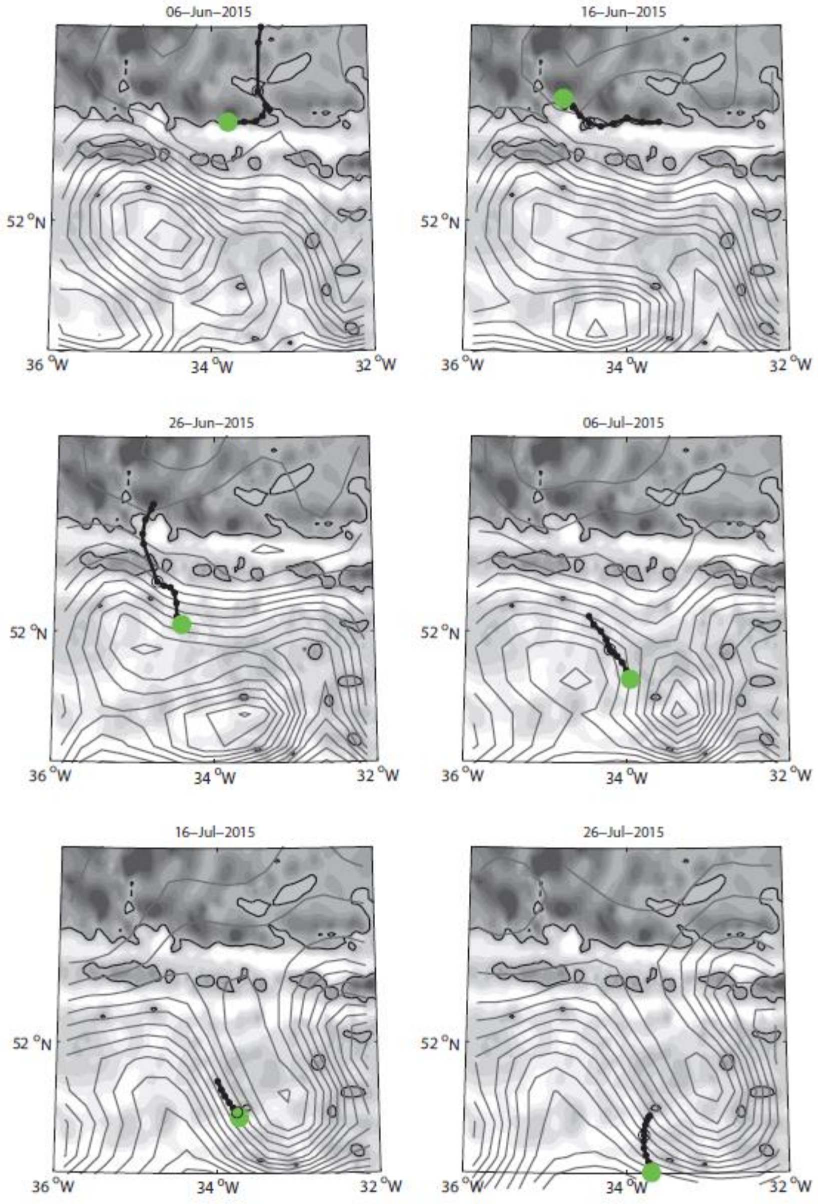

Supplementary Figure 2. **Interaction between the eddies/meanders and a RAFOS float trajectory.** Time sequence of absolute dynamic topography (ADT; black contours) maps starting on 06-June-2015, with 10-day track segments for float 1326 superimposed. Black dots on the float tracks are daily. Contour interval for ADT is 2 cm. The 2000m and 3000m isobaths are shown in gray.

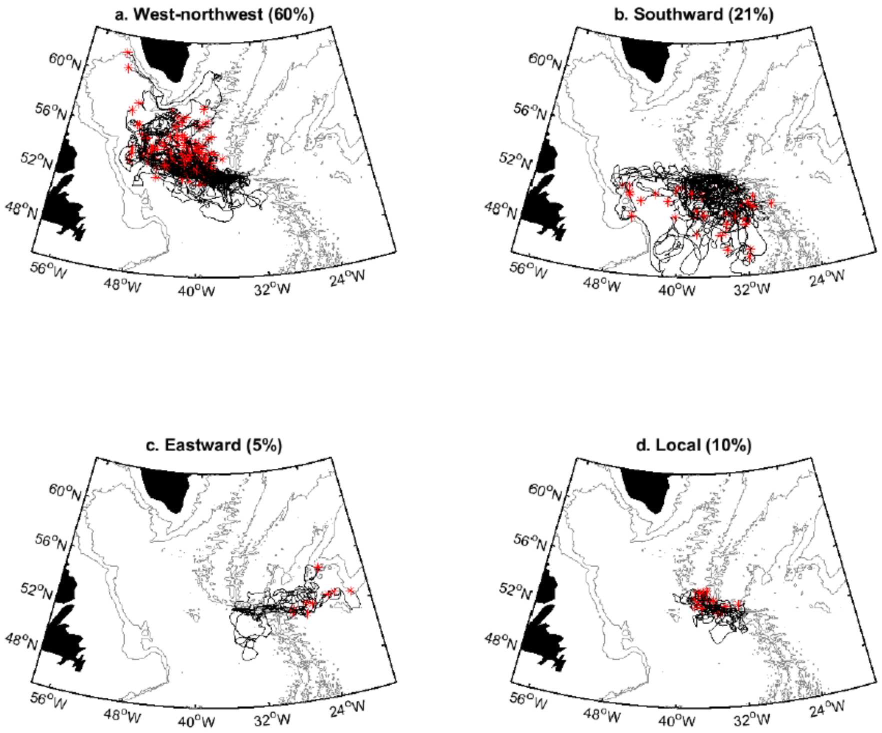

Supplementary Figure 3. An example of the simulated ISOW pathways. For clarity, only 200 floats were randomly selected to make these plots. The trajectories for each category are shown in black lines and the final locations of the floats in two years are indicated with red asterisks. The percentages of floats that take different pathways are also reported.

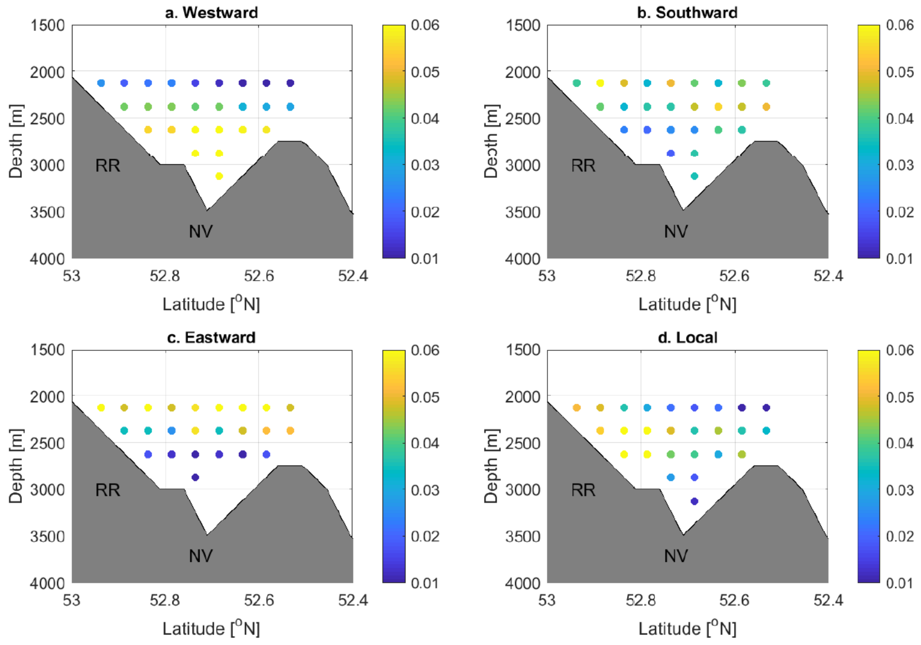

Supplementary Figure 4. Probability distribution of the initial launch locations for simulated floats taking different pathways. (a) For the 2151 floats that take the west-northwestward pathway. A larger value on a latitude-depth grid indicates that the westward travelling floats are more likely initialized at

this location. **(b-d)** Similar to **(a)**, but for 677 southward-flowing floats, 230 eastward-flowing floats and 331 locally-circulated floats. Acronyms in the figure: RR-Reykjanes Ridge; NV: northern valley.

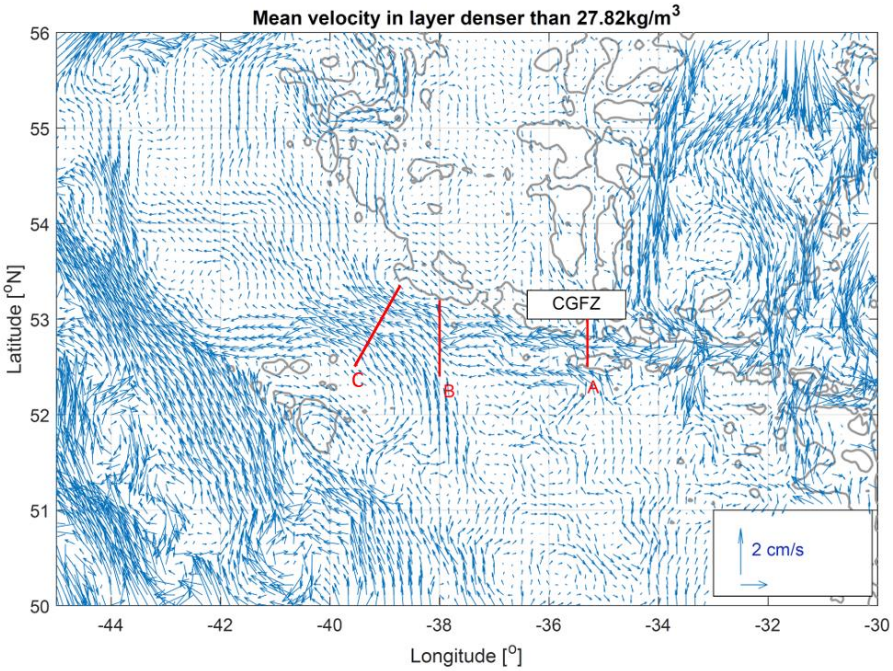

Supplementary Figure 5. **Simulated deep velocity field near the Charlie-Gibbs Fracture Zone (CGFZ).** Blue arrows show the 15-year mean velocity vectors averaged in the ISOW layer (denser than 27.82 $kg\ m^{-3}$). A west-northwestward deep mean current is present west of the CGFZ. The mean volume transport across the northern valley of the CGFZ (i.e. section A) is -0.6Sv with an annual standard deviation of 0.4 Sv. West of the CGFZ, the ISOW from the southern valley merges with that from the northern valley, resulting in an increased volume transport (-1.1±0.4Sv at 38°W; section B). Further west, another branch of deep waters flow northward from ~52ºN and turn cyclonically to join the westward spreading ISOW (section C). This branch seems to be a separation of the deep recirculation branch east of the deep western boundary current. The total transport across section C is -1.6±0.6 Sv.

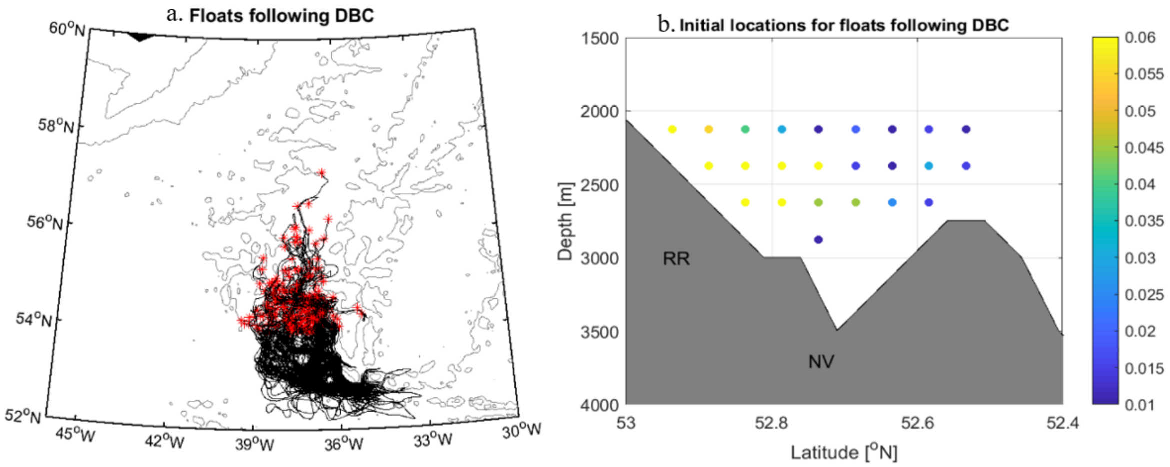

Supplementary Figure 6. **The northward spreading of the simulated floats. (a)** Trajectories of 201 simulated floats (total: 3593) that followed the deep boundary current (between 2000m and 3000m isobaths) continuously into the Irminger Sea. The floats' final locations after 2-year integration are

indicated with red asterisks. **(b)** Probability distribution of the initial launch locations for the 201 floats in the Charlie-Gibbs Fracture Zone. Acronyms in the figure: RR-Reykjanes Ridge; NV: northern valley.

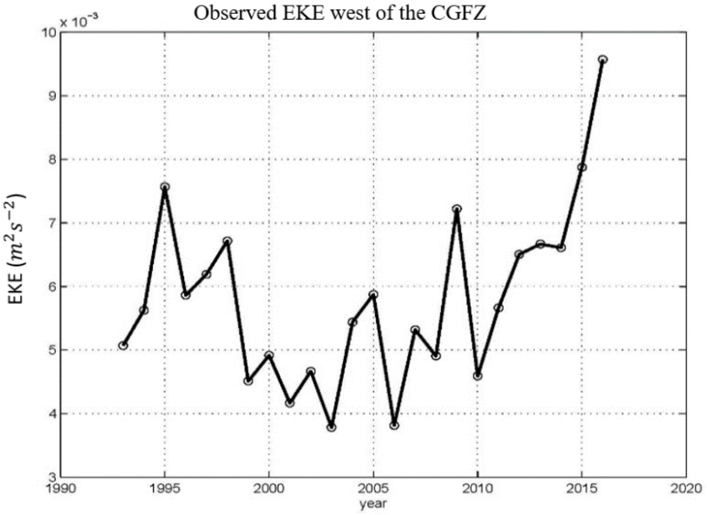

Supplementary Figure 7. Observed time series of eddy kinetic energy (EKE) west of the Charlie-Gibbs Fracture Zone. The EKE is calculated with daily geostrophic current anomaly product from AVISO (https://www.aviso.altimetry.fr/en/home.html) over the box [40ºW, 34ºW] and [51ºN, 54ºN]. The daily EKE is then averaged annually. The time period of 2014-2016 is characterized as a period with relatively high EKE.

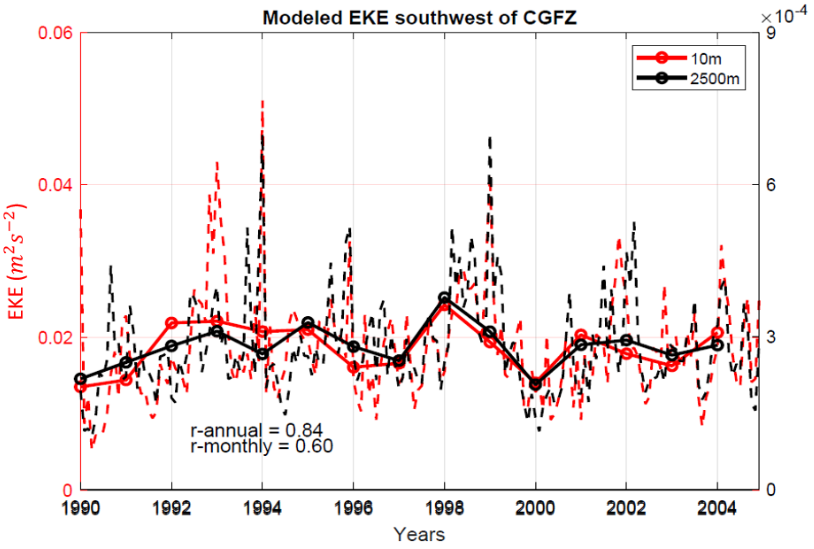

Supplementary Figure 8. **Simulated time series of eddy kinetic energy (EKE) west of the Charlie-Gibbs Fracture Zone (CGFZ).** Red (black) lines show the time series of 10m (2500m) EKE averaged over the box southwest of CGFZ (**Figure 4b**) in FLAME. The EKE at each grid is computed as $(u'^2 + v'^2)/2$, where u' and v' are the velocity anomalies relative to the 15 year mean. Dashed lines indicate the monthly time series while solid lines represent the annual means. A linear trend has been removed for all time series. The 15 year mean 10m (2500m) EKE is 0.018 ($2.8 \times 10^{-4} m^2 s^{-2}$), with an annual standard deviation (STD) of 0.003 ($0.4 \times 10^{-4} m^2 s^{-2}$) . The 10m EKE and 2500m EKE are significantly correlated both on monthly and interannual time scales (at 99% confidence level based on T-test).

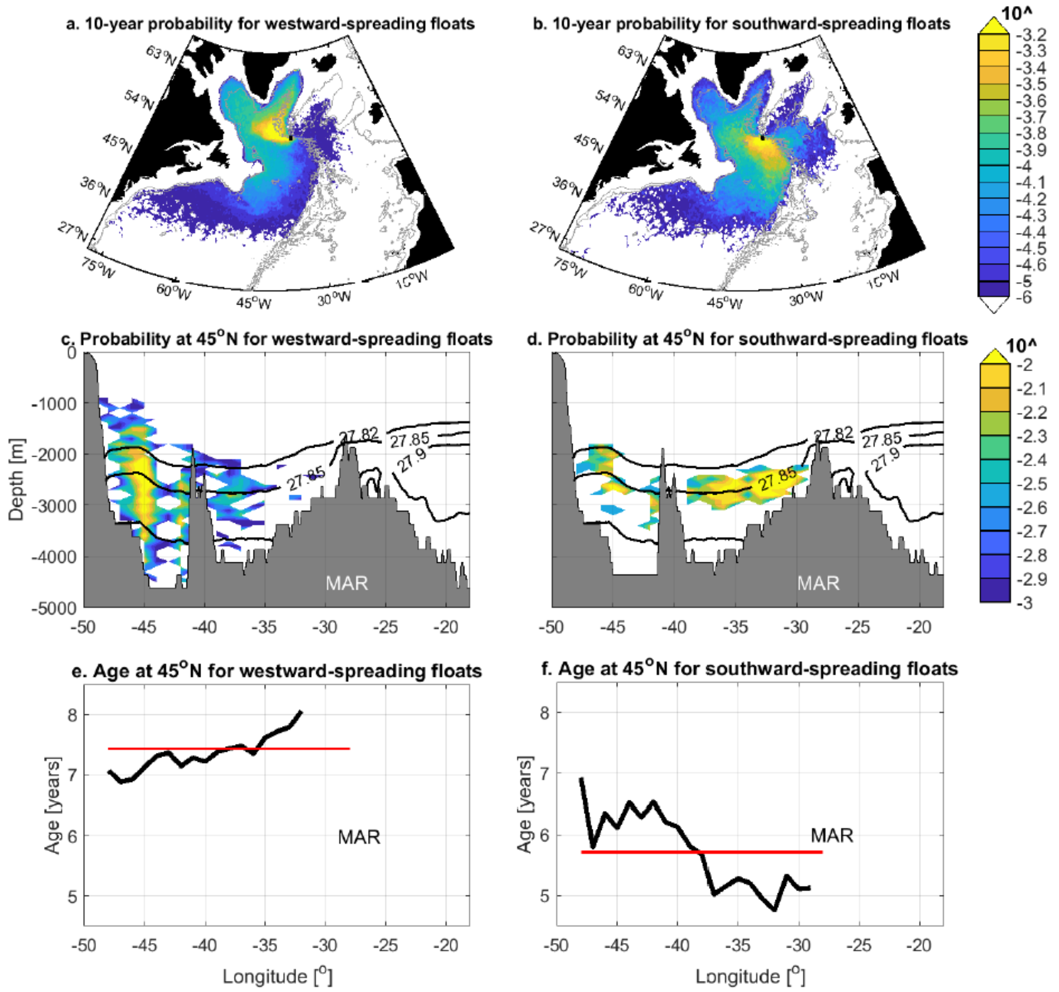

Supplementary Figure 9. Simulated spreading pathways of ISOW over 10 years. (a,c,e) Plots for 2151 (total: 3593) floats that follow the west-northwestward pathway during their first 2 years. **(a)** Probability map of these float trajectories after 10-year integration. **(c)** Probability distribution of the floats' first occurrences at 45ºN within 10 years. **(e)** Age of the floats' first occurrences at 45°N as a function of longitude (solid black line). The averaged age across all longitudes is indicated as a red solid line (7.5 years). **(b,d,f)** Similar to the left panels, but for 677 floats that follow the southward pathway during their first 2 years. **(b)** Probability map of 10-year float trajectories. **(d)** Probability distribution of floats' first occurrences at 45ºN. **(f)** Age of the floats' first occurrences at 45°N. The averaged age is 5.7 years and is shown with a red line. All probability plots are on log scale.

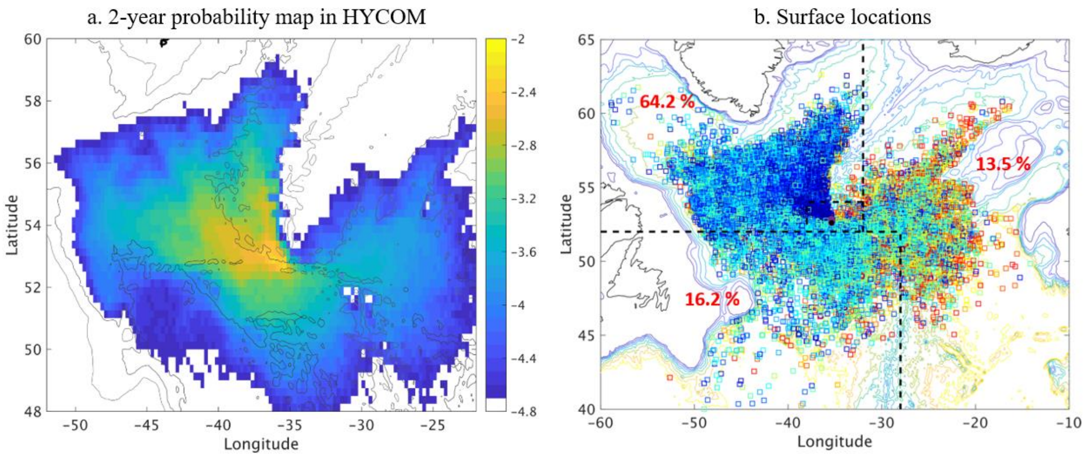

Supplementary Figure 10. Simulated ISOW spreading pathways in model HYCOM. (a) Probability distribution of float trajectories integrated along isopycnals based on a 1/12º Atlantic simulation using HYCOM. The details of the simulation and the modeled circulation in the subpolar North Atlantic, including the ISOW properties and volume transport near the Charlie-Gibbs Fracture Zone, are documented in Xu et al[1,2]. The numerical floats were released every 10 days in three ISOW layers that centered near 2100, 2500, and 2800m (σ_2 of 36.97, 37.02 and 37.06 $kg\ m^{-3}$) from 1990 to 2015 and were integrated forward for two years basin on daily mean outputs. **(b)** Surface locations of all the 22,464 floats two years after launch. The percentage in the box denotes the west-northwestward, southward and eastward pathways.

Supplementary References

1. Xu, X. et al. On the currents and transports connected with the atlantic meridional overturning circulation in the subpolar North Atlantic. *Journal of Geophysical Research: Oceans*, 118, 502-516 (2013).
2. Xu, X., Bower, A., Furey, H. & Chassignet, E. Variability of the Iceland-Scotland Overflow Water Transport Through the Charlie-Gibbs Fracture Zone: Results From an Eddying Simulation and Observations. *Journal of Geophysical Research: Oceans*, 123, 5808-5823 (2018).
